# Supplementary material for: Current Situation for Pharmacists in Japanese Veterinary Medicine: Exploring the Pharmaceutical Needs and Challenges of Veterinary Staff to Facilitate Collaborative Veterinary Care
Source: Pharmacy (Basel). 2024 Nov 29;12(6):179. doi: 10.3390/pharmacy12060179 (PMC11677796; doi:10.3390/pharmacy12060179)
Supplement: Supplementary file 1 [file pharmacy-12-00179-s001.zip › Figure S1.pdf]

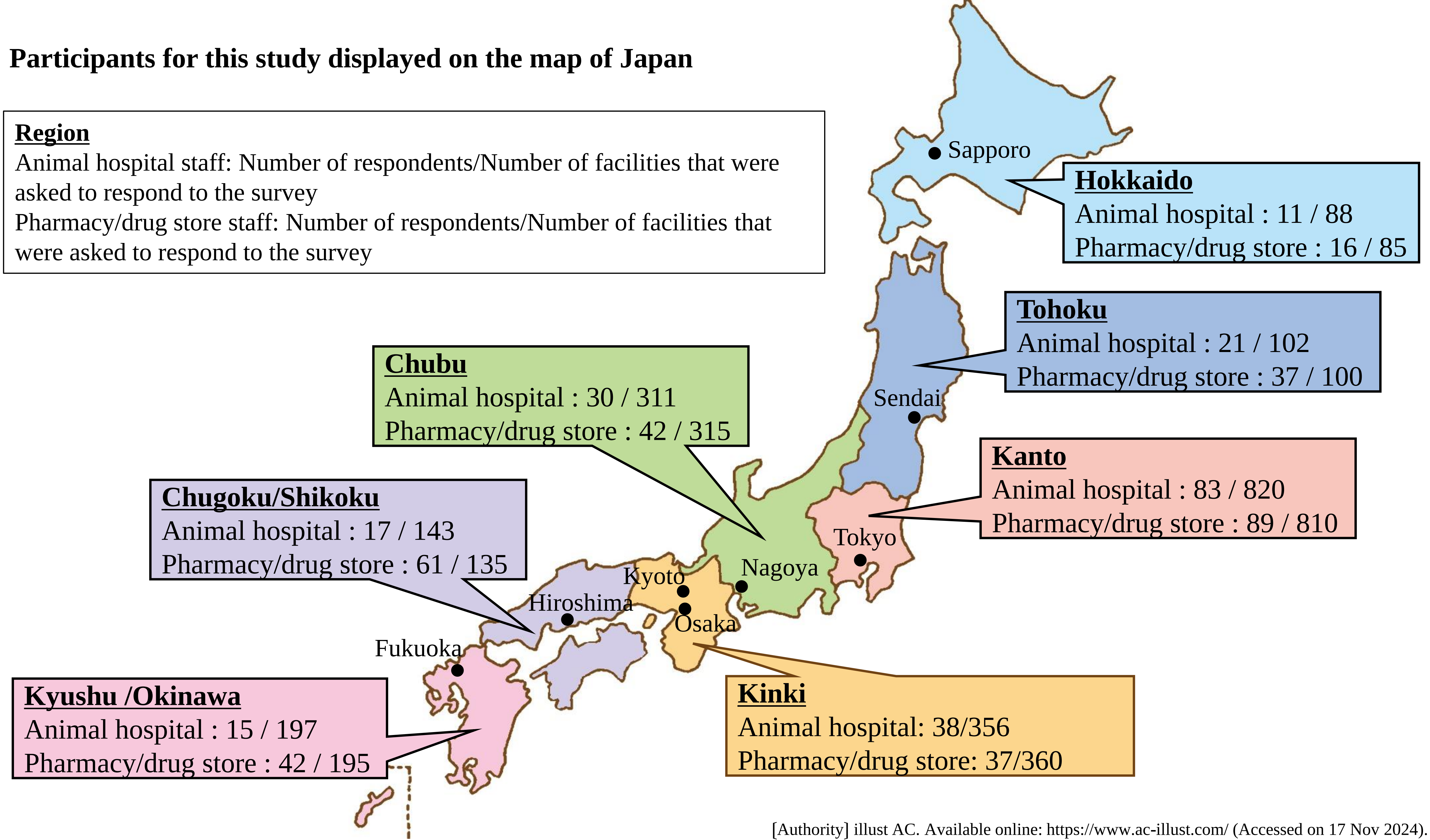
Participants for this study displayed on the map of Japan
Region
Animal hospital staff: Number of respondents/Number of facilities that were asked to respond to the survey
Pharmacy/drug store staff: Number of respondents/Number of facilities that were asked to respond to the survey
Sapporo
Hokkaido
Animal hospital : 11 / 88
Pharmacy/drug store : 16 / 85
Tohoku
Animal hospital : 21 / 102
Pharmacy/drug store : 37 / 100
Sendai
Chubu
Animal hospital : 30 / 311
Pharmacy/drug store : 42 / 315
Kanto
Animal hospital : 83 / 820
Pharmacy/drug store : 89 / 810
Tokyo
Chugoku/Shikoku
Animal hospital : 17 / 143
Pharmacy/drug store : 61 / 135
Nagoya
Kyoto
Hiroshima
Osaka
Fukuoka
Kyushu /Okinawa
Animal hospital : 15 / 197
Pharmacy/drug store : 42 / 195
Kinki
Animal hospital: 38/356
Pharmacy/drug store: 37/360
[Authority] illust AC. Available online: https://www.ac-illust.com/ (Accessed on 17 Nov 2024).
