# Supplementary material for: Current Situation for Pharmacists in Japanese Veterinary Medicine: Exploring the Pharmaceutical Needs and Challenges of Veterinary Staff to Facilitate Collaborative Veterinary Care
Source: Pharmacy (Basel). 2024 Nov 29;12(6):179. doi: 10.3390/pharmacy12060179 (PMC11677796; doi:10.3390/pharmacy12060179)
Supplement: Supplementary file 1 [file pharmacy-12-00179-s001.zip › File S1.pdf]

Supplementary Material, Questionnaire S1

	**Questionnaire Survey Regarding the Involvement of Pharmacists in Small-Animal Clinical Practice for Pharmacies and Drug Stores**	
**No.**	**Contents of question**	**Answer choices**
1	If you do not consent to this study, you will not be able to participate. Please make sure you fully understand the purpose of this study and select whether or not you consent.	[1] I agree to participate in the research; [2] I do NOT agree to participate in the research
	**1) Respondent's basic information**	
**No.**	**Contents of question**	**Answer choices**
1	Age (years)	[1] 20s; [2] 30s; [3] 40s; [4] 50s; [5] 60s; [6] ≥ 70s
2	Affiliation	[1] Pharmacies (whose main business is dispensing prescriptions); [2] Kampo pharmacies; [3] Drug stores; [4] Companies handling veterinary drugs; [5] Others
3	Region	[1] Hokkaido; [2] Tohoku; [3] Kanto; [4] Chubu; [5] Kinki; [6] Chugoku/Shikoku; [7] Kyushu/Okinawa
4	Number of employees at your facility (per facility)	Free comment
5	Is there a veterinary hospital near your institution (in front of the gate, within approximately 50–100 m)?	[1] Yes; [2] No
6	Years with pharmacist's license	Free comment
7	If you have a medical certification, please enter the name of that certification.	Free comment
8	If you have a veterinarian license, veterinary nurse for companion animals license, or other private	Free comment

	qualifications related to animals please enter those qualifications.	
	**2) Actual situation and pharmaceutical challenges related to pharmacist duties in veterinary medicine**	
**No.**	**Contents of question**	**Answer choices**
1	Please select the answer that applies to the following regarding “Pharmacists being involved (including the possibility that they may be able to) in veterinary and companion animals medical care.”	[1] I have experience thinking about this; [2] I do NOT have experience thinking about this; [3] I cannot choose between the options
2	Have you ever been asked by a veterinarian to dispense or prepare a medicine (including a prescription or verbal instructions, etc.)?	[1] Yes; [2] No<br>※If you selected “Yes,” please provide information such as 1) the number and frequency of prescriptions you received, 2) a rough outline of the prescription, and 3) your relationship with the prescribing facility (whether you know them, distance from the facility, background, etc.).
3	Have you ever prepared any medication based on a prescription (including oral instructions) from a veterinarian?	[1] Yes; [2] No<br>※If you selected “Yes,” please provide specific details about what you did to respond to the consultation.
4	Have you ever been consulted or asked questions about companion animal medicines or drug therapy from a patient?	[1] Yes; [2] No<br>※If you selected “Yes,” please provide specific details.
5	Have you ever received consultations or questions about veterinary medicine or drug therapy from veterinary staff?	[1] Yes; [2] No<br>※If you selected “Yes,” please provide specific details.
6	When you have visited a veterinary hospital, have you ever wondered	[1] Yes; [2] No; [3] I have never been to a veterinary hospital

	about any questions from a pharmacist's perspective about the dispensing, drug preparation, drug sales, etc. that are carried out at the animal medical facility?	※If you selected "Yes," please provide specific details.
7	When you visit a veterinary hospital, what do you think about the medication instructions and information provided from a pharmacist's perspective?	[1] Very satisfied; [2] Somewhat satisfied; [3] Neither satisfied nor dissatisfied; [4] Somewhat dissatisfied; [5] Very dissatisfied; [6] Never visited a veterinary hospital<br>※If you selected an option other than "Never visited a veterinary hospital," please provide the reason for your answer.
8	When you visited a veterinary hospital, have you ever been aware of a pharmaceutical-related accident or incident?	[1] Yes (both accidents and incidents); [2] Yes (accidents); [3] Yes (incidents); [4] No; [5] I do not know if I have experience or not; [6] Never visited a veterinary hospital
9	Please select all medicines, health foods, etc. that your facility handles and that have been dispensed or sold for the purpose of treating or promoting the health of pets.	[1] Not used; [2] Medical drugs for humans (prescription required); [3] Chinese medicine for humans; [4] Over-the-counter drugs for humans; [5] Health foods for humans; [6] Supplements for humans; [7] Veterinary drugs (prescription required); [8] Chinese medicine for animals; [9] Over-the-counter drugs for animals; [10] Health foods for animals; [11] Supplements for animals<br>※If you dispensed any products, please provide the product name.
10	It is said that 80–90% of pharmaceuticals used in clinical practice for small animals are human medicines. Article 23 of the Pharmacists Act stipulates dispensing of pharmaceuticals	[1] Yes; [2] No<br>※If you selected "No," please state the reason. If your answer was "Yes," please be specific about how you would respond.

	based on prescriptions from veterinarians, but if your facility receives a prescription from a veterinarian, are you currently able to dispense or prepare the medicine?	
	**3) Possibility of pharmacist involvement in promoting team-based veterinary care**	
**No.**	**Contents of question**	**Answer choices**
1	Looking ahead to the promotion of team veterinary medicine, would you like to work with veterinarians and veterinary nurses for companion animals in the future?	[1] I strongly agree; [2] I think so; [3] Neither agree nor disagree; [4] I do not think so; [5] I definitely do not think so<br>※Please enter the reason for your answer.
2	With an eye toward promoting team veterinary care, please answer each question about the contributions you think pharmacists can make to the promotion of team veterinary care.<br><br>12 choices: (a) prepare the drugs with a prescription (excluding injectable drugs), (b) prepare the injectable drugs, (c) formulation preparation of medicines used in the hospital, (d) confirmation of prescription contents and dispensing medicines, (e) mixing and preparation of pharmaceuticals, (f) medication guidance, (g) drug information service (for veterinary staff), (h) drug information service (for pet owners, but excluding medication guidance), (i) sales of over-the-counter drugs and	[1] Able to contribute a lot; [2] If anything, able to contribute somewhat; [3] Neutral position; [4] Somewhat unable to contribute; [5] Unable to contribute at all<br>※Please enter any other work or matters that could contribute to companion animal medicine.

	supplements, (j) drug management guidance work, (k) therapeutic drug monitoring, (l) pharmaceutical management operations	
3	Are there any obstacles for pharmacists to participate in team veterinary care?	[1] Yes; [2] No; [3] I cannot choose an option<br>※If you selected “Yes,” please provide specific details.
4	In promoting team veterinary care, do you have any requests for veterinary medical personnel or veterinary medical facilities from your standpoint as a pharmacist?	[1] Yes; [2] No<br>※If you selected “Yes,” please provide specific details.
5	If there are any learning opportunities such as information exchanges, study or training sessions with veterinarians, animal medical facilities, or people involved in veterinary medicine, please answer whether or not you would like to participate.	[1] I definitely want to participate; [2] I would rather participate; [3] I cannot choose an option; [4] I would rather not participate; [5] I definitely do not want to participate<br>※If you answered “I definitely want to participate” or “I would rather participate,” what kind of theme would you like?
6	When you receive questions or consultations from veterinary medical staff, what is your own attitude toward responding to them? Also, how confident are you about the answer you selected? Please select the closest match choices.	(a) I will decline all requests or consultations, regardless of their content; (b) I will listen to the questions or consultations, determine whether I can respond, and only reply to those that I can answer immediately; (c) I will respond within the scope of my knowledge, regardless of the content of the question or consultation, including those that I cannot answer immediately; (d) I will respond within the scope of my knowledge, regardless of the content of the question or consultation, including those I cannot answer immediately. Additionally, for matters I am unsure about, I will seek guidance

		while respecting and understanding the other person's expertise to provide an informed response and advice; (e) I will not know until I am in that situation
7	How do you think the involvement of pharmacists in small animal clinical practice will change veterinary medicine in the future? Select all that apply.	(a) Veterinarians will be able to demonstrate their professional skills better; (b) Veterinary nurses for companion animals will be able to demonstrate their professional skills better; (c) Pharmacists will be able to demonstrate their professional skills better; (d) Veterinary staff will be able to perform their daily work more efficiently; (e) The quality of veterinary care is expected to improve; (f) Drug therapy outcomes will improve; (g) The number of accidents and incidents will increase; (h) The number of accidents and incidents will remain the same; (i) The number of accidents and incidents will decrease; (j) Owner satisfaction will increase; (k) Owner satisfaction will remain the same; (l) Owner satisfaction will decrease; (m) Separation of medical and pharmaceutical services will progress; (n) Separation of medical and pharmaceutical services will not progress; (o) Veterinary hospital management will improve; (p) Veterinary hospital management will worsen; (q) No change even with the involvement of pharmacists; (r) Owners' medical expenses will increase; (s) Owners' medical expenses will remain the same; (t) None of these apply; (u) I cannot choose an option; (v) Others<br>※If you selected "Others," please provide specific details.

8	When pharmacists are involved in companion animals' clinical practice, how do you think they should acquire knowledge about veterinary medicine and animals? Please select all choices closest to your notion.<br><br>13 choices: (a) Not sure where to start studying ; (b) Proceed with the necessary learning and knowledge acquisition under the guidance of a veterinarian; (c) Participate in veterinary medical societies and seminars; (d) Acquire the necessary knowledge while gaining clinical experience at a pharmacy where you can be involved in companion animal treatment; (e) Self-study using the internet and specialized books (for veterinarians); (f) Self-study using the internet and specialized books (for veterinary nurses for companion animals); (g) Collect and aggregate the latest knowledge from papers related to veterinary medicine; (h) Acquire the necessary knowledge while gaining clinical experience at veterinary hospitals; (i) Aim to obtain a veterinary nurse for companion animals license; (j) Aim to obtain a veterinary license; (k) Incorporate	[1] Most applicable; [2] Appropriate to some extent; [3] Neither agree nor disagree; [4] Somewhat does not apply; [5] Does not apply<br>※If you selected "Other," please enter the details.

	the topic into the pharmaceutical education curriculum; (l) Launch an academic society for pharmacists involved in veterinary medicine to share information; (m) Other	
	**4) Free description section**	
**No.**	**Contents of question**	**Answer choices**
1	If you have any opinions, thoughts, or suggestions about this research, please enter them in the free-form text field.	Free comment
