# Supplementary material for: Current Situation for Pharmacists in Japanese Veterinary Medicine: Exploring the Pharmaceutical Needs and Challenges of Veterinary Staff to Facilitate Collaborative Veterinary Care
Source: Pharmacy (Basel). 2024 Nov 29;12(6):179. doi: 10.3390/pharmacy12060179 (PMC11677796; doi:10.3390/pharmacy12060179)
Supplement: Supplementary file 1 [file pharmacy-12-00179-s001.zip › File S2.pdf]

Supplementary Material, Questionnaire S2

	**Questionnaire Survey Regarding the Involvement of Pharmacists in Small-Animal Clinical Practice for Animal Hospitals**	
**No.**	**Contents of question**	**Answer choices**
1	If you do not consent to this study, you will not be able to participate. Please make sure you fully understand the purpose of this study and select whether or not you consent.	[1] I agree to participate in the research; [2] I do NOT agree to participate in the research
	**1) Respondent's basic information**	
**No.**	**Contents of question**	**Answer choices**
1	Age (years)	[1] 20s; [2] 30s; [3] 40s; [4] 50s; [5] 60s; [6] ≥ 70s
2	Affiliation	[1] Animal hospital (university hospital); [2] Animal hospital (corporate management); [3] Animal hospital (individual management)
3	Region	[1] Hokkaido; [2] Tohoku; [3] Kanto; [4] Chubu; [5] Kinki; [6] Chugoku/Shikoku; [7] Kyushu/Okinawa
4	Number of employees at your facility (per facility)	Free comment
5	Is there a pharmacy near your institution (in front of the gate, within approximately 50–100 m)?	[1] Yes; [2] No
6	Years of veterinarian/veterinary nurse for companion animals/pharmacist licensure	Free comment
7	If you have a medical certification, please enter the name of that certification.	Free comment
	**2) Actual situation and pharmaceutical challenges related to pharmacist duties in veterinary medicine**	
**No.**	**Contents of question**	**Answer choices**

1	Please select the answer that applies to the following regarding “Pharmacists being involved (including the possibility that they may be able to) in veterinary and companion animals medical care.”	[1] I have experience thinking about this; [2] I do NOT have experience thinking about this; [3] I cannot choose between the options
2	Does your animal hospital currently have a pharmacist?	[1] Not enrolled; [2] Currently enrolled (one person); [3] Currently enrolled (two people); [4] Currently enrolled (more than three people); [5] There used to be a pharmacist, but they are absent now; [6] Double-license holders enrolled (veterinarian/pharmacist); [7] Double-license holders enrolled (veterinary nurse for companion animals/pharmacist)
3	In human medicine, pharmacists perform the following tasks – who is primarily responsible for these tasks at your hospital?<br><br>12 choices: (a) prepare the drugs with a prescription (excluding injectable drugs), (b) prepare the injectable drugs, (c) formulation preparation of medicines used in the hospital, (d) confirmation of prescription contents and dispensed medicines, (e) mixing and preparation of pharmaceuticals, (f) medication guidance, (g) drug information service (for veterinary staff), (h) drug information service (for pet owners, but excluding medication guidance), (i) sales of over-the-counter	[1] Veterinarian; [2] Veterinary nurse for companion animals; [3] Pharmacist; [4] Outsourcing; [5] General employee; [6] Person other than [1] to [5]; [7] Not applicable/not performing the job

	drugs and supplements, (j) drug management guidance work, (k) therapeutic drug monitoring, (l) Pharmaceutical management operations	
4	When performing work related to medicines and drug therapy, do you have any problems, doubts, concerns, uncertainties, or trouble making decisions?	[1] Yes; [2] No<br>※If you selected “Yes,” please provide specific details.
5	Please select the choices that apply to your hospital’s issuance of prescriptions	[1] No prescriptions issued (in-hospital dispensing); [2] Issuing prescriptions (outside hospital dispensing, *excluding outsourcing); [3] Prescriptions may be issued (in-hospital and out-of-hospital dispensing); [4] Outsourcing the process from issuing prescriptions to dispensing
6	Please select the choices that apply to your animal hospital’s visitors’ use of the companion animal medicine notebook	[1] Approximately 90% or more of visitors use it; [2] Approximately 70% of visitors use it; [3] Approximately 50% of visitors use it; [4] Approximately 30% of visitors use it; [5] Approximately 10% of visitors use it; [6] There is no notebook
7	Please select the choice that applies to your animal hospital’s distribution status of drug information documents (explanatory documents regarding prescription drugs)	[1] Issued to everyone; [2] Issued to those who request; [3] Not issued
8	Do you have experience using anti-cancer drugs (both for humans and animals)? (*Experience refers to all work-related aspects such as inventory management, preparation,	[1] Yes; [2] No<br>※If you selected “Yes,” please select all the anti-cancer drugs you have used in the past.<br>(a) Cyclophosphamide; (b) Ifosfamide; (c) Melphalan; (d) Temozolomide; (e)

	administration, and drug administration guidance)	Procarbacin; (f) Chlorambucil; (g) Nimustine; (h) Lomustine; (i) Methotrexate; (j) Fluorouracil; (k) Cytarabine; (l) Gemcitabine; (m) Hydroxycarbamide; (n) L-Asparaginase; (o) Doxorubicin hydrochloride; (p) Mitoxantrone; (q) Hydrochloride; (r) Bleomycin; (s) Actinomycin D; (t) Epirubicin hydrochloride; (u) Vincristine sulfate; (v) Vinblastine sulfate; (w) Paclitaxel; (x) Cisplatin; (y) Carboplatin; (z) Other
9	Do you have experience using molecular targeted drugs (both for humans and animals)? (*Experience refers to all work-related aspects such as inventory management, preparation, administration, and drug administration guidance)	[1] Yes; [2] No<br>※If you selected "Yes," please select all the anti-cancer drugs you have used in the past.<br>(a) Imatinib; (b) Dasatinib; (c) Vemurafenib; (d) Dabrafenib; (e) Trametinib; (f) Encorafenib; (g) Binimetinib; (h) Masitinib; (i) Toceranib; (j) Oclacitinib; (k) Other
10	Please select all that apply regarding your post-graduate education and learning regarding the above-mentioned anti-cancer drugs and molecular targeted drugs.	[1] Attended training sessions hosted by veterinary medical societies; [2] Attended training sessions hosted by veterinary associations; [3] Attended training sessions hosted by pharmaceutical/medical device manufacturers for animals; [4] Attended training sessions hosted by human medical societies; [5] Attended training sessions hosted by medical associations; [6] Attended training sessions hosted by pharmaceutical/medical device manufacturers for humans; [7] Collected information from specialist books; [8] Collected information from academic papers; [9] Collected information from package

		inserts; [10] Attended training at other training facilities; [11] Other
11	When using anti-cancer drugs and molecular targeted drugs, do you have any problems, doubts, concerns, things you do not understand, or are unsure about (or anything you need to be careful about)?	[1] Yes; [2] No<br>※If you selected “Yes,” please provide specific details.
12	Have you ever been aware of a pharmaceutical accident or incident?	[1] Yes (both accidents and incidents); [2] Yes (accidents); [3] Yes (incidents); [4] No; [5] I do not know if you have experience or not
13	When you come across something you do not understand about medicines or drug therapy, where do you gather and what information do you use?	Free comment
	**3) Possibility of pharmacist involvement in promoting team-based veterinary care**	
**No.**	**Contents of question**	**Answer choices**
1	Looking ahead to the promotion of team veterinary medicine, would you like to work with pharmacists in the future?	[1] I strongly agree; [2] I think so; [3] neither agree nor disagree; [4] I do not think so; [5] I definitely do not think so<br>※Please enter the reason for your answer.
2	With an eye toward promoting team veterinary care, please answer each question regarding the tasks you would like to request from a pharmacist.<br><br>12 choices: (a) prepare the drugs with a prescription (excluding injectable drugs), (b) prepare the injectable drugs, (c) formulation preparation of medicines used in the hospital, (d) confirmation of prescription contents and dispensed medicines, (e) mixing	[1] I want to actively request; [2] If anything, I would like to request; [3] Neutral position; [4] I would rather not request; [5] I never want to request; [6] Already done by pharmacists; [7] Not performing this job<br>※If you have any other duties that you would like the pharmacist to perform, please enter them in detail.

	and preparation of pharmaceuticals, (f) medication guidance, (g) drug information service (for veterinary staff), (h) drug information service (for pet owners, but excluding medication guidance), (i) sales of over-the-counter drugs and supplements, (j) drug management guidance work, (k) therapeutic drug monitoring, (l) Pharmaceutical management operations	
3	Are there any obstacles for pharmacists to participate in team veterinary care?	[1] Yes; [2] No; [3] I cannot choose from the options<br>※If you selected "Yes," please provide specific details.
4	In promoting team veterinary care, do you have any requests for pharmacists or pharmacies from your standpoint as an animal hospital staff member?	[1] Yes; [2] No<br>※If you selected "Yes," please provide specific details.
5	If there are any learning opportunities such as information exchanges, study or training sessions with pharmacists, pharmacies, or pharmaceutical professionals, please answer whether or not you would like to participate.	[1] I definitely want to participate; [2] I would rather participate; [3] I cannot choose an option; [4] I would rather not participate; [5] I definitely do not want to participate<br>※If you answered "I definitely want to participate" or "I would rather participate," what kind of theme would you like?
6	Would you like access to an environment where you can feel free to ask questions and consult with pharmacists when you have questions or do not understand something related to pharmaceuticals?	[1] I strongly agree; [2] I think so; [3] Neither agree nor disagree; [4] I do not think so; [5] I definitely do not think so
7	How do you think the involvement of	[1] Veterinarians will be able to demonstrate

	pharmacists in small-animal clinical practice will change veterinary medicine in the future? Select all that apply.	their professional skills better; [2] Veterinary nurses for companion animals will be able to demonstrate their professional skills better; [3] Pharmacists will be able to demonstrate their professional skills better; [4] Veterinary staff will be able to perform their daily work more efficiently; [5] The quality of veterinary care is expected to improve; [6] Drug therapy outcomes will improve; [7] The number of accidents and incidents will increase; [8] The number of accidents and incidents will remain the same; [9] The number of accidents and incidents will decrease; [10] Owner satisfaction will increase; [11] Owner satisfaction will remain the same; [12] Owner satisfaction will decrease; [13] Separation of medical and pharmaceutical services will progress; [14] Separation of medical and pharmaceutical services will not progress; [15] Veterinary hospital management will improve; [16] Veterinary hospital management will worsen; [17] No change even with the involvement of pharmacists; [18] Owners' medical expenses will increase; [19] Owners' medical expenses will remain the same; [20] None of these apply; [21] I cannot choose an option; [22] Others<br>※If you selected "Others," please provide specific details.
8	When pharmacists are involved in companion animal clinical practice, how do you think they should acquire knowledge about veterinary medicine	[1] Most applicable; [2] Appropriate to some extent; [3] Neither agree nor disagree; [4] Somewhat does not apply; [5] Does not apply<br>※If you selected "Other," please enter the

	and animals? Please select the one that is closest to your notion.<br><br>13 choices: (a) Not sure where to start studying ; (b) Proceed with the necessary learning and knowledge acquisition under the guidance of a veterinarian; (c) Participate in veterinary medical societies and seminars; (d) Acquire the necessary knowledge while gaining clinical experience at a pharmacy where you can be involved in companion animal treatment; (e) Self-study using the internet and specialized books (for veterinarians); (f) Self-study using the internet and specialized books (for veterinary nurses for companion animals); (g) Collect and aggregate the latest knowledge from papers related to veterinary medicine; (h) Acquire the necessary knowledge while gaining clinical experience at veterinary hospitals; (i) Aim to obtain a veterinary nurse for companion animals license; (j) Aim to obtain a veterinary license; (k) Incorporate the topic into the pharmaceutical education curriculum; (l) Launch an academic society for pharmacists involved in veterinary medicine to share information; (m) Other	details.
9	Do you have any hopes or plans for	[1] As we have a pharmacist on staff, we have

	hiring new pharmacists in the future?	no plans to hire anyone; [2] Currently employed: dual license holder (veterinarian/pharmacist); [3] Currently employed: dual license holder (animal nurse/pharmacist); [4] Currently, we do not have a pharmacist, and we have no plans or desire to hire anyone in the future; [5] Currently, we do not have a pharmacist, but we do have plans and desire to hire someone in the future; [6] Currently, we plan to have a dual license holder (veterinarian/pharmacist) in the future; [7] Currently, we plan to have a dual license holder (animal nurse/pharmacist) in the future; [8] Pharmacist work has already been outsourced or prescriptions issued for external use; [9] We are considering outsourcing pharmacist work or issuing prescriptions for external use in the future; [10] Others
10	[Pharmacists Only]<br>Please provide your thoughts on 1) the needs and future prospects for pharmacists in small-animal clinical practice and 2) how pharmacists should acquire specialized knowledge about animals when working in small-animal clinical practice, taking into account your own experiences.	Free comment
11	[Only for those with veterinarian/pharmacist licenses (double license)]<br>Please provide your thoughts, based on your own experiences, on 1) the need	Free comment

	for and future prospects for pharmacists in small-animal clinical practice and 2) how pharmacists should acquire specialized animal knowledge when working in small-animal clinical practice.	
	**4) Free description section**	
**No.**	**Contents of question**	**Answer choices**
1	If you have any opinions, thoughts, or suggestions about this research, please enter them in the free-form text field.	Free comment
