# Supplementary material for: Current Situation for Pharmacists in Japanese Veterinary Medicine: Exploring the Pharmaceutical Needs and Challenges of Veterinary Staff to Facilitate Collaborative Veterinary Care
Source: Pharmacy (Basel). 2024 Nov 29;12(6):179. doi: 10.3390/pharmacy12060179 (PMC11677796; doi:10.3390/pharmacy12060179)
Supplement: Supplementary file 1 [file pharmacy-12-00179-s001.zip › File S3.pdf]

**Supplementary Material S4. Respondent Characteristics**

**Table S1.** Respondents' age

| Characteristic | | N (%) | Total |
|---|---|---|---|
| Animal hospital staff | 20s | 13 (6.0) | 217 |
| | 30s | 50 (23.0) | |
| | 40s | 67 (30.9) | |
| | 50s | 56 (25.8) | |
| | 60s | 24 (11.1) | |
| | ≥ 70s | 6 (2.8) | |
| | No answer | 1 (0.4) | |
| Pharmacy and drug store staff | 20s | 26 (8.0) | 324 |
| | 30s | 110 (34.0) | |
| | 40s | 83 (25.6) | |
| | 50s | 63 (19.4) | |
| | 60s | 31 (9.6) | |
| | ≥ 70s | 10 (3.1) | |
| | No answer | 1 (0.3) | |

**Table S2.** Respondents' license years

| Characteristic | | | N (%) | Total |
|---|---|---|---|---|
| Animal hospital staff | Veterinarian | 1–5 | 4 (2.2) | 181 |
| | | 6–10 | 22 (12.2) | |
| | | 11–20 | 54 (29.8) | |
| | | 21–30 | 64 (35.4) | |
| | | 31–40 | 28 (15.5) | |
| | | ≥ 41 | 8 (4.4) | |
| | | No answer | 1 (0.5) | |
| | Veterinary nurse for companion animals | 1–5 | 6 (17.6) | 34 |
| | | 6–10 | 12 (35.3) | |
| | | 11–20 | 12 (35.3) | |
| | | 21–30 | 1 (3.0) | |
| | | 31–40 | 3 (8.8) | |
| | Pharmacist | 1–5 | 1 | 2 |
| | | 21–30 | 1 | |
| Pharmacy and drug store staff | Pharmacist | 1–5 | 42 (13.0) | 324 |
| | | 6–10 | 64 (19.8) | |
| | | 11–20 | 76 (23.4) | |
| | | 21–30 | 76 (23.4) | |
| | | 31–40 | 45 (13.9) | |
| | | ≥ 41 | 19 (5.9) | |
| | | No answer | 2 (0.6) | |
